# Supplementary material for: Identification and Characterization of p300-Mediated Lysine Residues in Cardiac SERCA2a
Source: Int J Mol Sci. 2023 Feb 9;24(4):3502. doi: 10.3390/ijms24043502 (PMC9959367; doi:10.3390/ijms24043502)
Supplement: Supplementary file 1 [file ijms-24-03502-s001.zip › ijms-2142986-supplementary.pdf]

Supplementary Materials

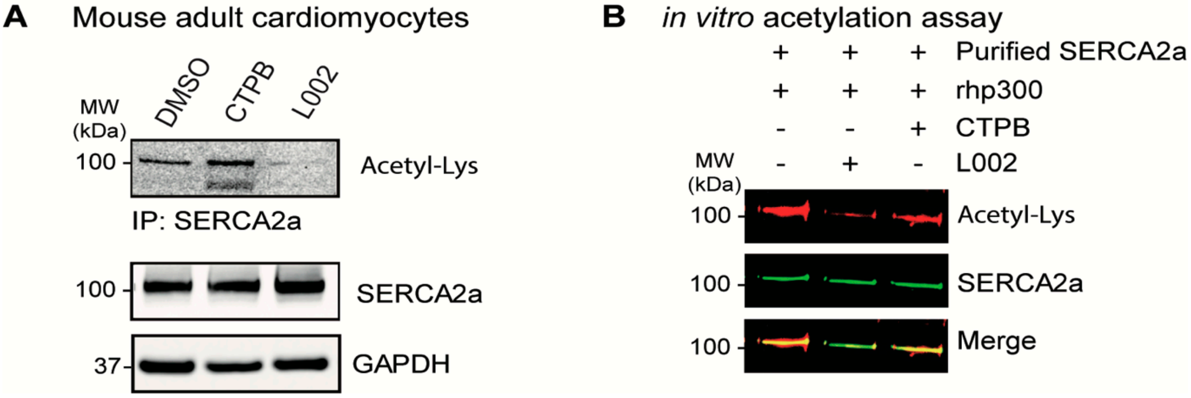

Supplemental Figure S1. Regulation of p300 activity by small molecules changes SERCA2a acetylation. A. Mouse adult cardiomyocytes (ACMs) were isolated from male C57BL/6J mice and treated with 5 μM CTPB (p300 activator) or 1 μM L002 (p300 inhibitor). 24 hours after treatment with CTPB or L002, the SERCA2a acetylation was assessed by immunoprecipitation with anti-SERCA2a and detected by immunoblotting with anti-Acetyl-Lys antibody. B. Purified porcine SERCA2a protein was incubated with hp300 (human recombinant p300, rhp300) protein in the presence of CTPB or L002. The acetylation status of purified porcine SERCA2a protein was confirmed by immunoblotting with anti-Acetyl-Lys antibody.
